# Ensuring Equitable Access to the COVID-19 Vaccine: The Experience of A Local Health Unit in Rome, Italy

**DOI:** 10.3390/healthcare10112246

**Published:** 2022-11-10

**Authors:** Federica Turatto, Michele Sassano, Mauro Goletti, Santino Severoni, Adriano Grossi, Paolo Parente

**Affiliations:** 1 Department of Public Health and Infectious Diseases, Sapienza University of Rome, 00185 Rome, Italy; 2 Department of Life Science and Public Health, Section of Hygiene, Institute of Public Health, Università Cattolica del Sacro Cuore, 00168 Rome, Italy; 3 Local Health Authority ASL Roma 1, 00193 Rome, Italy; 4 Health and Migration Programme, World Health Organization, 1211 Geneva, Switzerland

**Keywords:** COVID-19, vaccination, health equity, access to healthcare, public health, allocation

## Abstract

Growing evidence is emerging on the higher risk of infection and adverse outcomes for the most disadvantaged groups of the population, and COVID-19 vaccination campaigns worldwide are struggling to ensure equitable access to immunization for all. From 21 June 2021 to 15 October 2021, the Local Health Unit ASL Roma 1 adopted a tailored immunization strategy to reach socially vulnerable groups of the population with the primary vaccination course. This strategy was developed with a step-by-step, participatory approach. Through engagement with internal and external stakeholders, target groups were identified, potential barriers analyzed, solutions discussed, and tailored interventions designed. Over nine thousand individuals from among irregular migrants, homeless people and hard-to-reach communities were contacted and vaccinated.

## 1. Introduction

As of August 2022, more than two years since its emergence, the SARS-CoV-2 pandemic has led to approximately 578 million cases and over 6.4 million deaths and still represents an enormous challenge for health systems worldwide [1]. After an initial outbreak reported in Wuhan (Hubei, China) in December 2019, COVID-19 spread rapidly across countries and was declared a pandemic by WHO on 11 March 2020 [2]. The symptoms of the SARS-CoV-2 infection include dry cough, fever and fatigue, and its manifestations range from asymptomatic or mild cases to severe cases resulting in hospitalization and death [3]. To contain the spread of the virus and reduce the negative effects on the health of the population, drastic measures were put in place including lockdowns, social isolation and travel bans, which have had grave economic and social effects, leading to an unprecedented global crisis [4,5,6,7,8]. Moreover, the pandemic has had important effects on mental health, especially among children and adolescents [9,10]. Available data point to a disproportionate impact of this crisis on the most fragile groups among the population, thus contributing to increased socio-economic inequalities [11,12,13,14,15]. However, despite the restrictive measures adopted by countries worldwide, the virus is still circulating and affecting daily life. In order to effectively tackle the COVID-19 emergency, the need for vaccine development catalysed the efforts of the scientific community, leading to effective vaccines becoming available less than one year after the virus was identified. Starting in December 2020, the mRNA vaccines developed by Pfizer/BioNTech and Moderna and the viral vector vaccines developed by Oxford-Astrazeneca and Janssen received authorization for emergency use, allowing vaccination campaigns to start worldwide [16,17].

The need to immunize the entire population against the virus, however, has raised questions regarding their allocation criteria, both locally and globally [18,19,20,21,22]. According to guidelines from the World Health Organization (WHO), in supply-constrained situations the immediate objective of the vaccination strategy is to reduce mortality and reduce the pressure on the health systems and should thus address population groups that present a greater risk of infection and negative clinical outcomes, such as the elderly, extremely fragile individuals, patients affected by chronic conditions, and healthcare workers. As immunization programs progress, the choice of the following target populations should be guided by criteria that consider not only epidemiological evidence, but also economic, social and ethical requirements [23].

In this context, the need to ensure equitable vaccine distribution with specific attention to the socially disadvantaged and hard-to-reach groups of the population has been highlighted [19,23,24,25]. In fact, socially vulnerable individuals, including ethnic minorities, homeless people, migrants in reception centres, and people with limited access to health services, have been identified as categories at a higher risk of infection and worse health outcomes [26,27,28,29,30,31,32,33,34]. Immunizing these categories is of utmost importance to ensure an equitable response to the pandemic, and also contributes to the success of the vaccination campaign and limiting the spread of the virus, as the housing and working conditions of these population groups often lead to a greater risk of infection and might facilitate occurrence of epidemic clusters [30,35].

Thus, evidence is required to develop equitable access strategies for COVID-19 vaccination. This paper describes the experience of the Local Health Unit (LHU) ASL Roma 1 in providing equitable access to the COVID-19 vaccine to socially vulnerable individuals from 21 June 2021 to 15 October 2021. The ASL Roma 1 is located in the northern half of Rome, in the Lazio Region, in central Italy, and manages access to healthcare for almost 1 million inhabitants. It is composed of different Units under the supervision of the Health Directorate and operates through six Health Districts. These Health Districts provide access to healthcare services and ensure coordination and continuity in response to the health needs of the population through integration with primary care and hospital care. In Italy, the LHUs play a fundamental role in organizing and managing the anti-SARS-CoV-2 vaccination campaign, working in close contact with the Regional Health Directorates.

## 2. National and Regional Regulatory Context

The immunization of socially vulnerable individuals, the core activity herein described, has been partially addressed by regional and national policies and guidelines, which provide the framework for the general indications followed in designing these activities.

In Italy, as in other European countries, the vaccination campaign against SARS-CoV-2 officially kicked-off on 27 December 2020 [36]. The National Strategic Plan for anti-SARS-CoV-2 vaccination issued on 2 January 2021 identified healthcare workers, nursing home guests and staff members, and older people as priority groups. As the immunization campaign progressed, on 10 March 2021, new national guidelines were issued and priority groups were redefined to include progressively younger age groups, especially those considered fragile based on their health conditions. For the first time, non-health-related social characteristics were also considered, leading to school staff, the security forces, prison detainees and individuals residing or working in residential communities being defined as priority groups for vaccination [37]. In addition, the Lazio Region further indicated that these priority groups should also include workers and residents of communities hosting socially vulnerable individuals, such as minors, individuals with disability or psychiatric disorders, and women facing difficulties [38].

However, as the vaccination campaign progressed, access to vaccination for migrants, homeless people and hard-to-reach populations remained an unsolved issue. Despite recognizing the entitlement to vaccination for every individual present in Italy regardless of their legal status, in most regions—including Lazio—the online reservation platform did not allow access for those not registered with the National Health Service (NHS) [39,40].

## 3. A Vaccination Strategy to Reach the Most Vulnerable

In the absence of national or regional guidelines specifically addressing this issue, the ASL Roma 1 developed an immunization strategy targeted at the most socially vulnerable categories of the population, which was carried out between June 2021 and December 2021.

The strategy was officially communicated to the Lazio Region on 16 June 2021. Regional and national guidelines were issued soon after: on the same day, the Lazio Region encouraged LHUs to develop strategies specifically addressed to the socially vulnerable and hard-to-reach categories of the population [41]. At the national level, on 18 June 2021, the Italian Ministry of Health clarified that the Janssen vaccine, initially recommended only for individuals over 60, could also be administered to non-sedentary individuals or those with high work mobility aged 60 or younger, and in general to all hard-to-reach population groups, due to the convenience offered by the single-dose vaccination schedule [42].

In addition, on 18 July 2021, the Italian National Institute of Health (ISS) released recommendations both for priorities and implementation of anti-SARS-CoV-2 vaccination in residential communities, which were in line with the strategy proposed and followed by the ASL Roma 1 in implementing the vaccination campaign [43].

### 3.1. Adapting the WHO Framework for Tailored Immunization Programmes

The immunization strategy developed by ASL Roma 1 was inspired by the Tailored Immunization Programme (TIP) framework set out by WHO, aimed at guiding countries in ensuring equity of vaccination programs for all population groups [44]. Given the extraordinary circumstances of the COVID-19 pandemic, this framework was adapted to comply with time and resource constraints while maintaining the values underpinning the TIP approach intact. Providing equitable access to and utilization of vaccination services was the main goal of the campaign, following nationally stated health goals related to vaccine coverage aimed at halting the spread of the pandemic. This strategy was developed using a people-centred, participatory method: stakeholder engagement was in fact a key aspect. Input from internal and external stakeholders at all levels was encouraged throughout the process to identify the most appropriate vaccination strategies. Community leaders were contacted to discuss vaccine intentions as well as other social and health needs of the community. Specific attention was given to involving Non-Governmental Organizations (NGOs), charities and associations working with those marginalized communities identified as key targets in this process, to provide vaccine-related information, given their ability to interact with the hardest-to-reach. Comprehensiveness in identifying barriers and drivers of vaccination behaviour is a key aspect of the WHO framework. The theoretical framework suggested is the Capability, Opportunity, and Motivation Model of Behaviour (COM-B model), which identifies behaviours as results of the interaction of factors related to individual capability and motivation, as well as opportunities offered by each specific context [45]. Analysing barriers to vaccination through focus groups with internal and external stakeholders was the first fundamental step in the vaccination strategy developed by ASL Roma 1, with specific attention given to structural barriers. Stakeholders analysed potential barriers starting from the theoretical framework of the COM-B model, thus focusing on factors influencing behaviour in the following domains: target population’s psychological (e.g., knowledge) or physical (e.g., skills) capability, social (e.g., societal influences) or physical (e.g., environmental resources) opportunity, and automatic (e.g., emotion) or reflective (e.g., beliefs, intentions) motivation [45,46]. Thus, mapping COM-B components facilitated the identification of strategies addressing relevant barriers for each component. This entire process was carried out on the basis of available scientific evidence, especially in defining high-risk hard-to-reach communities.

### 3.2. Developing the Vaccination Strategy

The definition of the vaccination strategy by ASL Roma 1 was organized into 3 steps:

- Step 1: Situation analysis and definition of targets
- Step 2: Analysis of potential barriers to immunization
- Step 3: Intervention design and implementation

**Figure 1:**
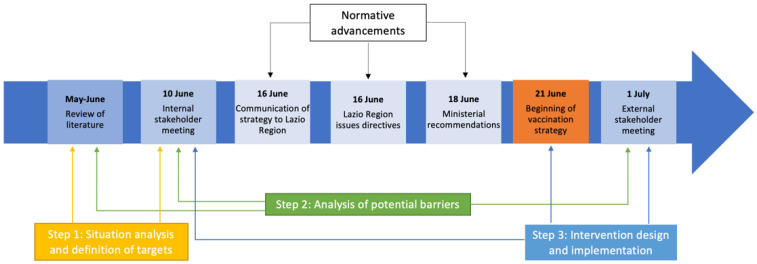
Timeline of the development of the vaccination strategy for socially vulnerable categories.

An overview of the main events is provided in Figure 1.

#### 3.2.1. Step 1: Situation Analysis and Definition of Targets

Identifying and defining the targets for this campaign was based on current scientific literature and on stakeholder engagement. The current national and international scientific literature highlights the need to include socially vulnerable categories in COVID-19 vaccination campaigns, with special attention given to the homeless and undocumented migrants [40,47,48]. Several NGOs and charities operating in the territory of the ASL Roma 1 also highlighted the issue, as such categories were at risk of being excluded from the vaccination campaign as initially designed at national level [49].

Various stakeholders at different levels of the ASL Roma 1 were also involved in elaborating available evidence and defining the targets. A TIP approach was deemed necessary for the following hard-to-reach categories among the targets of the strategy:

- Occupied buildings (facilities occupied by unauthorised individuals, families and social movements for residential purpose)
- Informal settlements
- Homeless people
- Undocumented migrants and others with limited access to the NHS

During the internal kick-off meeting held on June 10th, the initiative was shared with all stakeholders of the ASL Roma 1 and roles and responsibilities were defined.

#### 3.2.2. Step 2: Analysis of Potential Barriers to Immunization

An initial overview of the literature was conducted to identify potential barriers to vaccination for the target populations. Concerns about vaccine safety, scarce information, insufficient health literacy, lack of trust in health authorities, language barriers, lack of awareness about entitlement to vaccination and fear of being reported to the authorities were identified as potential barriers for migrants and homeless people [50,51,52,53].

Thanks to collaboration with internal and external stakeholders, such issues were discussed and the barriers to vaccination and how to overcome them were initially mapped by the ASL Roma 1 for each target category (Table 1). During the internal stakeholders meeting of 10 June 2021, the potential barriers and immunization drivers for each target were identified, then on 1 July 2021 an online kick-off meeting was held involving external stakeholders such as NGOs, charities and associations, who were encouraged to discuss barriers and drivers of vaccine behaviour for the population groups they represented.

From these meetings it emerged that priority had to be given to the structural barriers preventing access to the booking platform for vaccination for those not enrolled in the NHS. In order to address further barriers presented by motivational factors, on-site health promotional initiatives were designed together with community leaders. This analysis of barriers also led to selecting the Janssen vaccine for most of the targets, as it offers a complete vaccination cycle with a single shot: this choice was also supported by Ministerial recommendations which identified hard-to-reach populations as potential beneficiaries of the Janssen vaccine [42].

#### 3.2.3. Step 3: Intervention Design and Implementation

Based on the analysis of barriers to immunization uptake, specific strategies were developed for each target, which differed according to stakeholders involved, delivery sites and vaccine used. Local Health Districts, which often had pre-existing contacts with the communities targeted by the immunization strategy, played a major role in designing and rolling out these interventions.

##### Occupied Buildings

Pre-existing relationships, especially through social service officers of the ASL Roma 1, played a key role in programming and delivering anti-SARS-CoV-2 vaccination for occupied buildings. For each building, the leaders of these occupations were contacted to identify a common strategy to deliver the program. When deemed appropriate and feasible, tailored information campaigns were carried out prior to collecting consent for vaccination. Once lists were obtained, usually with the help of local community leaders, vaccine delivery was organized either on site or in nearby vaccination sites. The vaccine used for this target was usually the Janssen vaccine, due to its single-dose schedule.

##### Informal Settlements

The ASL Roma 1 had previously established contact with some *Roma* communities, both before or during the pandemic. Once again, building a relationship of trust with the health institutions was key to establishing a fruitful cooperation for the immunization campaign. Through the cooperation of community leaders, the ASL Roma 1 was able to obtain lists of people who agreed to be vaccinated and hence to implement the immunization program through on-site delivery of the Janssen and Pfizer vaccine, according to regional recommendations and availability of doses.

##### Homeless People, Undocumented Migrants and Others with Limited Access to the NHS

Reaching the homeless and undocumented migrants was the most challenging part of this campaign, as there was no one physical location where they could be reached. Therefore, in order to engage this sector of the population, different strategies were adopted. On the one hand, NGOs and associations working with migrants, homeless people or marginalized communities were contacted and involved in the process; because of their role as trusted contacts, they were able to effectively provide information about the vaccination campaign and collect consent statements. On the other hand, an “open night” was set up, during which people could be vaccinated without booking and without any legal status requirements [54]. In the meanwhile, an alternative online booking platform was developed for use by undocumented people unable to access the official regional booking website. This alternative platform was advertised and used both by individuals and by NGOs. Those included in this target were vaccinated in three vaccination hubs coordinated by ASL Roma 1, one of which was managed by an NGO. The vaccine administered was the Janssen vaccine. In these vaccination hubs, people were also encouraged to get a STP (Straniero Temporaneamente Presente) code, which allows foreigners who cannot legally register with the NHS (e.g., irregular migrants) to access healthcare for free [55].

## 4. Discussion

The pandemic has highlighted once again the importance of achieving high vaccination coverage to protect the health of the entire community. Ensuring equitable access to COVID-19 vaccines will therefore determine the success of the vaccination strategy, as well as the impact of the SARS-CoV-2 pandemic on health inequalities [56].

In Italy, specific action to ensure the immunization coverage of this population group was initiated months after the vaccination campaign had begun. Marginalization might explain the limited uptake of COVID-19 vaccinations among socially vulnerable individuals reported in the literature [30]. In fact, campaigns designed to deliver vaccination to the general population often fail to address issues pertaining to socially vulnerable individuals, who frequently face specific access barriers which require a tailored approach [44]. Emerging evidence seems to confirm that in hard-to-reach groups in Italy there is widespread acceptance of COVID-19 vaccination, reinforcing the need to remove structural barriers to vaccination. However, vaccine hesitancy appears to be higher compared to the general population in socially vulnerable population groups such as undocumented migrants and homeless people [57,58,59]. As indicated by previous reports, investing in tailored information campaigns, community involvement and outreach to tackle hesitancy seems thus key to ensuring that access to vaccination is truly equitable [60,61,62].

The TIP framework proposed by the WHO was used as a general guideline to program the vaccination campaign among socially vulnerable individuals. However, the TIP process was designed for normal circumstances, which is not the case of a global emergency such as the SARS-CoV-2 pandemic, and hence, strategies had to be adapted in order to achieve the goals within a reasonable timeframe.

Assessment of the effectiveness of the adopted strategy is beyond the scope of our paper, which aims to report our experience in the strategy development process. Hence, further research is needed to analyze the real-world effectiveness of the intervention, in order to assess the possibility of scaling up to other geographical and social contexts, and the potential to adapt and implement these strategies whenever appropriate.

## 5. Conclusions

This paper describes the development of a tailored vaccination strategy for the hard-to-reach groups of the population, providing evidence on potential interventions to promote vaccine equity during the COVID-19 pandemic. In such a complex and critical context, a coordinated and collaborative effort of all Units of the ASL Roma 1 together with local NGOs involved in the field of assistance to socially vulnerable individuals was crucial to reaching over 9000 individuals between June and October 2021, ensuring their primary course of vaccination. For many of them, this represented the first opportunity to interact with the NHS and to obtain an STP code which gives them access to a range of health services. Furthermore, the network of diverse actors involved in addressing the health and social needs of the most vulnerable was expanded and reinforced, laying the foundations for how best to address health inequalities in the future.

## Figures and Tables

**Table 1:** Analysis of barriers to vaccination and strategies to overcome them.

Target	Barriers	Strategies
Occupied buildings Informal settlements	Perceived marginality and isolation. Perceived discrimination by the institution. Lack of trust in health institutes.	Building trust in the institution by taking care of health and social needs. Health promotion interventions with on-site delivery of vaccine-related information. On-site delivery of the vaccine.
	Difficulty in scheduling second shot.	Use of Janssen vaccine.
Homeless people Undocumented migrants and other people with limited access to the NHS	Lack of legal requirements and/or technology to access the online vaccine booking platform.	Direct booking by the ASL based on consent statements collected by NGOs. Alternative online booking platform. Open night.
	Lack of information.	Delivering vaccine-related information through charities and NGOs.
	Difficulties in reaching vaccination hubs.	On-site delivery of the vaccine.
	Complex clinical situations. Communication difficulties during the anamnestic procedure. Linguistic barriers.	Training for healthcare personnel delivering the vaccine. Utilizing cultural mediators.
	Difficulty in scheduling second shot.	Use of Janssen vaccine.

## Data Availability

Not applicable.
